# THE ROLE OF LEADERSHIP IN DISASTER DECISION-MAKING IN ASSISTED LIVING

**DOI:** 10.1093/geroni/igad104.2050

**Published:** 2023-12-21

**Authors:** Lindsay Peterson, Debra Dobbs, David Dosa

**Affiliations:** University of South Florida, Tampa, Florida, United States; University of South Florida, Tampa, Florida, United States; Brown University, Providence, Rhode Island, United States

## Abstract

Assisted Living Communities (ALCs) make up a growing segment in long-term care for adults with impairments. Research indicates that functional and cognitively impaired older adults who are evacuated for hurricanes are more vulnerable to harm than those who shelter in place. A high proportion of assisted living residents require help with bathing (63.6%), dressing (48.2%), and other activities of daily living. At least 40% have Alzheimer’s disease or other dementias. Despite residents’ vulnerabilities, we know little about ALC disaster preparedness. In this study, we conducted a secondary analysis of data from interviews with ALC administrative staff after Hurricane Irma to better understand factors influencing the decision to evacuate or shelter in place. Initially we conducted individual interviews and focus groups with stakeholders of 70 Florida-based ALCs concerning a range of preparedness and response issues. We analyzed transcripts using a deductive approach, with research team members meeting regularly to resolve differences, develop/update codes, and identify themes and subthemes. Next, we separately analyzed codes concerning the decision to evacuate or shelter in place. Three overall themes were identified: 1) response to evacuation orders; 2) differing views of how to ensure safety; and 3) characteristics of ALC buildings. Examining our results overall, an overarching theme of leadership emerged, with effective leadership being preparedness based on comprehensive knowledge of residents’ needs, the nature of the threat at hand, and the ALC’s strengths and vulnerabilities. Our finding show the need for training incorporating “lessons learned” concerning both evacuation and sheltering in place for disasters.

